# Etiologies and treatments of chronic intestinal failure-short bowel syndrome (SBS) in Japanese adults: a real-world observational study

**DOI:** 10.1007/s00595-022-02469-9

**Published:** 2022-02-23

**Authors:** Tsunekazu Mizushima, Eri Udagawa, Miyuki Hasegawa, Yuko Tazuke, Hiroomi Okuyama, Jovelle Fernandez, Shiro Nakamura

**Affiliations:** 1 Osaka University Graduate School of Medicine, 2-2 Yamadaoka, Suita, Osaka 565-0871 Japan; 2 Takeda Pharmaceutical Company Limited, Tokyo, Japan; 3 Osaka Medical and Pharmaceutical University, 2-7 Daigakumachi, Takatsuki, Osaka 569-8686 Japan

**Keywords:** Intestinal failure, Short bowel syndrome, Parenteral nutrition, Intestinal failure-associated liver disease, Weaned off PN

## Abstract

**Purpose:**

Short bowel syndrome (SBS) with intestinal failure (SBS-IF) requires long-term parenteral nutrition (PN). This study investigated the real-world etiologies of SBS, treatment patterns, and PN-related outcomes among adult patients with SBS-IF in Japan.

**Methods:**

This retrospective, observational cohort study was based on data from April, 2008 to January, 2020 from one of the largest hospital-based claim databases in Japan. Analyzed patients were aged ≥ 16 years, had received continuous PN for ≥ 6 months, and had SBS or undergone SBS-related surgery with a diagnosis of a causative disease. The primary endpoint was PN weaning.

**Results:**

We analyzed data for 393 patients. The most frequent causes of SBS-IF were ileus (31.8%), Crohn’s disease (20.1%), and mesenteric ischemia (16.0%). Of 144/393 (36.6%) patients who were weaned off their PN, 48 (33.3%) were subsequently restarted on PN. Of 276/393 (70.2%) patients whose PN was initiated in hospital, 156 (56.5%) transitioned to home management. The mean duration of initial PN was 450.4 and 675.5 days for patients who were able or unable to be weaned off PN, respectively. Sepsis (67.4%), catheter-related bloodstream infections (49.1%), and liver disorders (45.0%) were the most reported PN-related complications.

**Conclusions:**

Most patients with SBS-IF in Japan could not be weaned off PN and suffered life-threatening complications.

**Supplementary Information:**

The online version contains supplementary material available at 10.1007/s00595-022-02469-9.

## Introduction

Short bowel syndrome (SBS) is a malabsorptive state caused by the physical or functional loss of portions of the small intestine. The leading cause of SBS in adults is extensive intestinal resection to manage Crohn’s disease (CD), cancer, mesenteric vascular disease, or traumatic injuries [1–4]. Data on the prevalence of SBS are limited globally and estimates vary by geographic region [2, 5, 6]. In a randomized nationwide survey conducted in Germany, 34 per million people were estimated to have SBS between 2011 and 2012 [6].

SBS is the most frequent pathophysiological mechanism of chronic intestinal failure (IF), defined as the reduction of gut function below the minimum necessary for the absorption of macronutrients and/or water and electrolytes, necessitating intravenous supplementation to maintain health and/or growth [7]. The mainstay of the nutritional management of patients with SBS is parenteral nutrition (PN), which can be given either at a hospital (inpatient or outpatient) or at home [8–10]. Patients with chronic IF associated with SBS (SBS-IF) become dependent on long-term PN, which is associated with risk of severe and/or chronic complications, reduced quality of life, and death [11–14]. Intestinal rehabilitation programs based on pharmacological treatment and surgical procedures facilitate intestinal adaptation, which could allow patients to wean off home PN (HPN) [11,15]. However, intestinal adaptation is a highly variable process unique to the individual patient [16]; therefore, weaning a patient off PN remains a challenge.

Comprehensive data on SBS-IF from analyses of large populations in Japan are lacking. A better understanding of the disease landscape in the real-world would contribute to the development of effective intestinal rehabilitation programs. This study used one of the largest hospital-based claim databases in Japan to reveal the etiologies of SBS, as well as the patterns of SBS-IF treatment in clinical practice and their outcomes.

## Methods

### Data source

This study used anonymized electronic health insurance claims and diagnosis procedure combination data provided by Medical Data Vision Co. Ltd. This large-scale, acute-care hospital-based database covers inpatient and outpatient data of 30.15 million patients from over 400 hospitals  [17]. The total population in Japan was 126 million as of 2020  [18]. The large population covered by the database allows rare diseases to be captured. Because the data were pre-existing and anonymized in an un-linkable manner, no ethical approval or patient consent was required for this study at the authors’ institutions, in accordance with Japan’s Ethical Guidelines for Epidemiologic Research. The data included information on the following: disease names coded using the World Health Organization’s International Statistical Classification of Diseases and Related Health Problems 10th Revision [ICD-10] coding scheme; and disease names, coded using Japanese Disease Name Codes, Medical Procedure Codes, and Medicine Codes for health insurance reimbursement. In the Japanese claim database, the diagnosis of SBS is identified by a disease receipt code of 8841646, as there is no specific ICD-10 code for SBS. Data used in this study covered the period from 1 April, 2008 to 31 January, 2020.

### Definitions

PN was defined as PN administered in any setting and HPN was defined as PN administered at home. PN in hospital was defined as PN administration in either an inpatient or outpatient setting. A PN episode was defined as a record of continuous PN for ≥ 6 months; the index PN event was defined as the first PN episode recorded in the database; and the index date was the date of initiation of the index PN event.

The time to first weaning was defined as days to first weaning from the index date, and total PN duration was the total duration of all PN episodes during the follow-up period. An index PN duration was defined as the duration of the index PN episode. Switching between PN at hospital and HPN was identified by two consecutive PN treatment records in the same month or in 2 consecutive months (Supplementary Fig. 1).

Up to 12 months before the index date was considered as the ‘lookback’ period to identify the cause of SBS-IF. If patients received their first PN infusion as a matter of urgency, most likely following an emergency event, the enrollment date and the index date were the same without a lookback period. The duration of follow-up after the index date was not predetermined; eligible patients were followed in the database for as long as they could be tracked. Patients who were transferred to another hospital for treatment during their index PN episode were excluded.

### Study population

Patients eligible for inclusion in the study were aged ≥ 16 years at the index PN event, had received continuous PN treatment for ≥ 6 months, and had reimbursement claims records for either SBS, or a causative disease of SBS (Supplementary Table 1) and surgery related to SBS (intestinal resection).

### Study endpoints

The primary endpoint of this study was PN weaning. Successful weaning was marked by intervals of > 2 months between PN records or intervals of > 2 months between the date of the last PN record and the last date of follow-up. Secondary endpoints included: PN restart, time to first weaning, index PN duration, total PN duration, transition to HPN, complications related to PN, duration of hospitalization, and number of recurrent hospitalizations. Information on the causative diseases of SBS-IF and patient comorbidities was also collected.

Ad hoc analyses were carried out to investigate patient characteristics, causes of SBS, PN-related complications (including deaths), discharge destination (outpatient, home, death, or other), and activities of daily living (ADLs).

### Statistical analysis

The study endpoints were analyzed using descriptive statistics. Continuous variables were reported using means and standard deviations (SDs) or medians and interquartile ranges, whereas categorical variables were reported using frequencies and percentages. All statistical analyses were conducted using SAS ver. 9.4 (SAS Institute; Tokyo, Japan).

Missing data were not imputed because of the descriptive analysis. Any patient with missing value(s) of outcome was included in the study population of baseline demographics; however, they were excluded from analyses that calculated the number and frequency of these outcomes with missing value(s).

## Results

### Patient characteristics

**Fig. 1:**
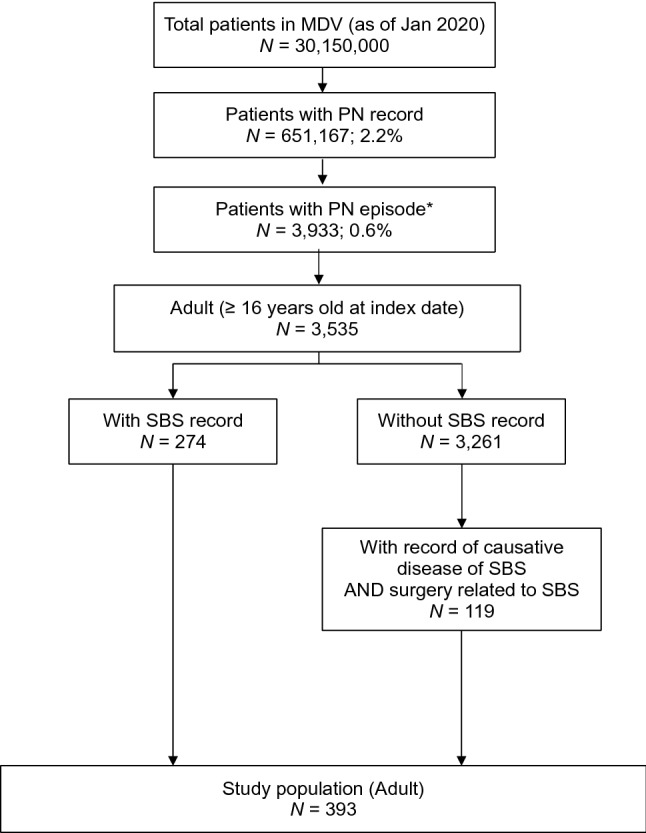
Patient selection flow chart. *A parenteral nutrition (PN) episode is defined as PN administration recorded continuously over ≥ 6 months. *MDV* Medical Data Vision, *PN* parenteral nutrition, *SBS* short bowel syndrome

Overall, 393 patients were eligible for inclusion in this study (Fig. 1). Of these, 274 (69.7%) had claims records for SBS and 119 (30.3%) patients had claims records for a causative disease of SBS and related surgery. The mean duration of the lookback period was 189.3 ± 163.7 days, and the follow-up period was 957 ± 786 days.

A comparable proportion of men and women were included in the study population (Table 1). At the PN index date, the mean age was 61.4 ± 17.3 years, and 58.6% of the patients were over 60 years of age. The mean body mass index (BMI) was 19.5 ± 4.5 kg/m^2^ for men and 18.5 ± 3.8 kg/m^2^ for women. The most frequent comorbidities were diabetes (36.4%) and hepatopathy (34.6%). The most common clinical departments attended by patients during the index hospitalization (*n* = 272) were general surgery (39.2%), gastroenterological surgery (8.4%), or internal medicine (7.6%) (Table 1). General surgery and gastroenterological surgery were reported separately as these are considered distinct specialties in Japan. Nearly half (46%) of the patients receiving index PN at a hospital (*n* = 272) had been referred from another hospital.

**Table 1:** Patient characteristics

Characteristic	*N* = 393
Sex, *n* (%)	
Male	205 (52.2)
Female	188 (47.8)
Mean age, years (SD)	61.4 (17.3)
Mean BMI, kg/m^2^ (SD)	
Male	19.5 (4.5)
Female	18.5 (3.8)
Causative diseases,^a^ *n* (%)	
Ileus (ICD-10: K567)	125 (31.8)
Crohn’s disease (ICD-10: K509)	79 (20.1)
Mesenteric ischemia (ICD-10: K55)	63 (16.0)
Others	61 (15.5)
Surgery related to SBS-IF,^b^ *n* (%)	
Colectomy	82 (20.9)
Small bowel obstruction surgery	63 (16.0)
Colostomy	48 (12.2)
Comorbidities, *n* (%)	
Diabetes	143 (36.4)
Hepatopathy	136 (34.6)
Dehydration	110 (28.0)
Malnutrition	62 (15.8)
Chronic kidney disease	52 (13.2)
Index hospitalization	272 (69.2)
Clinical department, *n* (%)	
General surgery	154 (39.2)
Gastroenterological surgery	33 (8.4)
Internal medicine	30 (7.6)
Others	55 (14.0)

*BMI* body mass index, *ICD-10* International Classification of Diseases, Tenth Revision, *SBS-IF* short bowel syndrome with intestinal failure, *SD* standard deviation

^a^Present in ≥ 10% of patients. A patient could record multiple causative diseases of SBS-IF

^b^Reported for ≥ 5% of patients

### Etiologies of SBS-IF

The top three causative diseases of SBS-IF were ileus (31.8%), CD (20.1%), and mesenteric ischemia (16.0%) (Table 1). The proportion of patients with CD receiving biologics increased from 25.4% before the first PN episode to 55.8% on the first episode (Supplementary Table 2).

The most commonly reported surgeries related to SBS were colectomy (20.9%), small bowel obstruction resection (16.0%), and colostomy (12.2%) (Table 1). A total of 173 (44.0%) patients underwent surgery in the same hospital as where they received their index PN.

### Weaning off PN and related outcomes

**Fig. 2:**
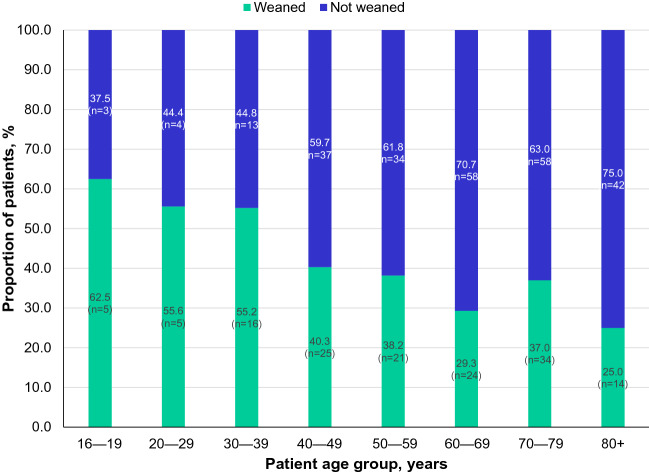
Rates of weaning from parenteral nutrition (PN) in the different patient age groups. *PN* parenteral nutrition

In total, 144 (36.6%) patients were weaned off PN; however, 48 (33.3%) of these 144 patients subsequently were restarted on PN. Weaning rates declined with increasing age, with lower rates observed in patients aged over 80 years (25.0%; 80–89 years [26.5%] and 90–99 years [14.3%]) than in younger age groups (Fig. 2). Overall, the mean age of patients who were weaned off PN was lower than that of patients who stayed on PN (57.8 ± 18.1 years vs 63.5 ± 16.4 years).

The mean index PN durations were 450.4 ± 339.7 and 675.5 ± 653.5 days for patients who were or were not able to be weaned off PN, respectively. The mean time to first PN weaning was 488.4 ± 356.3 days. Patients with claims records for SBS had over twice the mean index PN duration (714.3 ± 633.3 days vs 313.7 ± 188.7 days, respectively) and triple the mean total PN duration of patients without claims records for SBS (901.2 ± 757.8 days vs 326.9 ± 242.8 days, respectively) (Supplementary Fig. 2).

### Discharge destinations

Of the 276 (70.2%) patients who received PN at hospital for their index PN episode, 156 (56.5%) transitioned to HPN management during that episode. Of patients who had been hospitalized (*n* = 272), the mean duration of hospitalization was 170.9 ± 185.9 days. Forty-eight (17.6%) patients died during their index hospitalization. Patients who were transitioned from PN at hospital to HPN were generally younger than patients who were not (59.7 ± 16.4 years vs 71.4 ± 13.8 years, respectively). The mean number of recurrent hospitalizations was 4.3 ± 6.6.

Of the 120 patients who did not transition to HPN, 38.3% died, while another 27.5% were transferred to another hospital. Others were discharged to outpatient clinics (20.8% in the same hospital and 5.8% in a different hospital). Ileus and acute pan-peritonitis were the most reported causative diseases (32.5% and 16.7%, respectively) with the frequency of CD being low (2.5%). Ischemic heart disease (42.5%) and cerebrovascular disease (25.0%) were the leading illnesses potentially requiring continuous nursing care throughout the study period.

### Complications

**Fig. 3:**
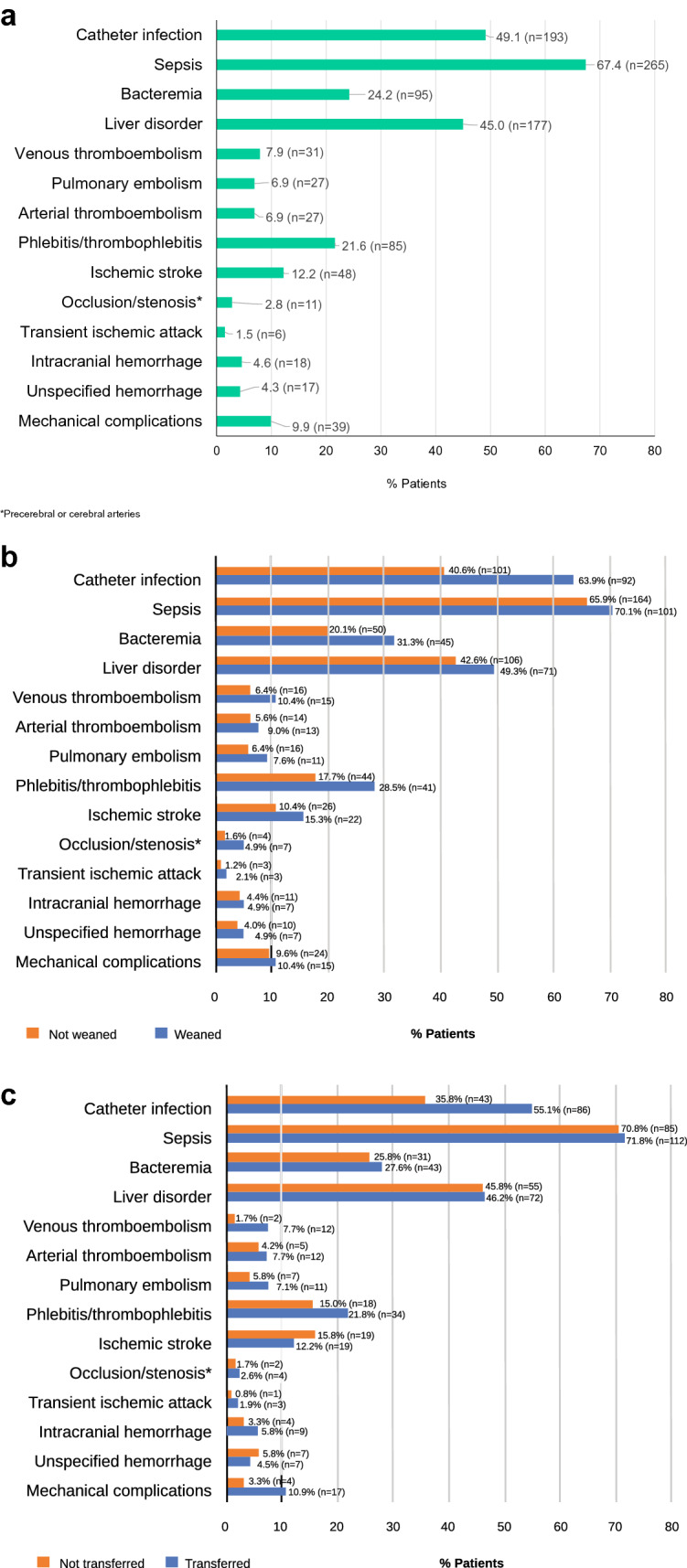
Complications related to parenteral nutrition (PN). **a** All complications; **b** Stratified by weaning from PN; **c** Transfer to home PN management. *Precerebral or cerebral arteries. *PN* parenteral nutrition

Of the total patient population (*n* = 393), sepsis, catheter-related blood infections, and liver disorders were the most common complications related to PN. Sepsis developed in 265 (67.4%) patients, catheter-related blood infections developed in 193 (49.1%), and subsequent liver disorders developed in 177 (45.0%) (Fig. 3).

The 48 patients who died during their index hospitalization had a higher mean age than the overall study population (72.8 ± 14.2 years vs 61.4 ± 17.3 years) and 90% of patients were aged > 60 years at the time of death. The main causes of SBS in these patients were ileus (37.5%) and acute pan-peritonitis (20.8%); CD accounted for only 2.1%. Catheter infection was the leading complication relating to PN but occurred less frequently in patients who died than in the overall population (27.1% vs 49.1%). Conversely, liver disorders occurred more frequently in patients who died than in the overall population (56.3% vs 45.0%) (Table 2). The mean time from index date to death was 308.0 ± 129.3 days.

**Table 2:** Complications associated with parenteral nutrition

Complications associated with parenteral nutrition	Proportion of total patients, *n* (%) (*N* = 393)	Proportion of patients who died, *n* (%) (*N* = 48)
Infectious complications		
Sepsis	265 (67.4)	34 (70.8)
Catheter infections	193 (49.15)	13 (27.1)
Bacteremia	95 (24.2)	12 (25.0)
Metabolic complications		
Liver disorders	177 (45.0)	27 (56.3)
Thromboembolism		
Phlebitis/thrombophlebitis	85 (21.6)	6 (12.5)
Ischemic stroke	48 (12.2)	7 (14.6)
Venous thromboembolism	31 (7.9)	2 (4.2)
Pulmonary embolism	27 (6.9)	1 (2.1)
Arterial thromboembolism	27 (6.9)	1 (2.1)
Occlusion or stenosis of precerebral or cerebral arteries	11 (2.8)	1 (2.1)
Transient ischemic attack	6 (1.5)	1 (2.1)
Intracranial hemorrhage	18 (4.6)	2 (4.2)
Unspecified hemorrhage	17 (4.3)	3 (6.3)
Mechanical complications	39 (9.9)	1 (2.1)

### Activities of daily living at discharge

**Fig. 4:**
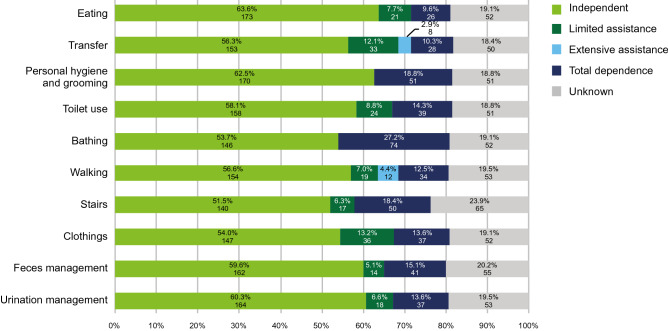
Scores for activities of daily living at the time of discharge

Two-thirds of patients (68.4%) were discharged from hospital during the study period. Figure 4 shows that most of these patients did not require assistance to carry out ADLs including eating, care of personal hygiene, toileting, and dressing, at the time of discharge from the index PN hospitalization.

## Discussion

This real-world study in Japan revealed that the most frequent causative diseases of SBS were ileus, CD, and mesenteric ischemia; consistent with previous global studies [2, 4]. The majority of adult patients with SBS-IF were unable to be weaned off PN dependency and older adults had lower weaning rates. Overall, only one-quarter of the patients were able to be weaned off PN effectively during the study period. Again, this result is consistent with previous studies reporting PN weaning rates of 20–50% for adult patients [2, 13, 16, 19].

The prevalence of CD was lower in the subgroups of patients who died or who did not transition to HPN during the study period (2.1–2.5% vs 20.1%). CD is typically diagnosed at age 15–35 years, hence, the patient population with CD may have been younger than the overall population. Furthermore, surgeries for ileus and mesenteric ischemia are more likely to be performed as emergency procedures, than surgeries for CD, suggesting possible differences in the underlying health of the patients receiving the respective surgeries. Finally, the use of biologics may have allowed the patients with CD to preserve residual bowel and improve their function, facilitating earlier weaning from PN. The increase in the use of biologics observed in this study may reflect the increase in availability of new biologics.

The top three complications associated with PN at hospital were sepsis, catheter-related blood infections, and liver disorders. Complications were common in patients who withdrew from PN, with approximately 50% experiencing life-threatening complications, such as sepsis and liver disorders. These results are consistent with those from a previous Japanese study on patients using central venous catheters (including patients with SBS), which found sepsis, phlebitis, infections, and inflammatory reactions to be common complications [20]. Most of the patients on PN at hospital in the present study transitioned to HPN. Those who remained on PN at hospital were older and more likely to have serious comorbidities. Nevertheless, the majority were discharged, and most were able to care for themselves and walk and climb stairs unaided. This is important, given that the need for assistance with ADL is associated with poor quality of life [21].

Forty-eight (12%) patients died during the study period. Other studies have reported a mortality rate of 12.8–50% [2, 15]; however, these rates may not be directly comparable because of the differences in study designs. The frequency of liver disorders was higher in patients who died than in the overall study population. Given the design of the present study, it is probable that the patients who died during the follow-up period had severe underlying comorbidities such as hepatic dysfunction before the index PN hospitalization. However, a previous report also suggested that liver complications in patients with SBS might not be linked to HPN but may instead result from the underlying IF [2].

The prevention and/or timely treatment of complications is a key strategy for both the survival and successful intestinal rehabilitation of patients with SBS [22]. For this reason, patients with SBS-IF, including those who have weaned off PN or have switched to HPN management, should be monitored carefully for signs of the onset of complications.

## Limitations

First, the study cohort was identified from records of reimbursement claims for SBS and we were unable to validate the patients’ clinical outcomes such as by means of chart reviews. To increase the accuracy of patient identification, we defined patients with SBS-IF as not only having claims records for SBS (or related diseases/procedures), but also as receiving long-term PN. Second, the database used in the study covers 23% of acute care hospitals in Japan, which are mainly larger hospitals (≥ 200 beds). Although patients with SBS-IF often receive care from larger hospitals with specialists, the findings of this study may not be inclusive of patients receiving care from smaller hospitals in Japan. Third, the database does not contain information from clinical examinations, such as the length of the remaining small intestine, which may affect outcomes, or any surgical records of advanced procedure for bowel lengthening, such as serial transverse enteroplasty or small bowel transplantation. Lastly, the analysis does not take into account any changes and developments in the medical system, including improvements on diagnostic tests, policy, or treatment in Japan during the study period of 2008–2020. Further studies may clarify details, including the weaning rate by causative disease and improvements in future treatment strategies.

## Conclusion

To our knowledge, this is the first study to provide comprehensive data on the causes and outcomes of SBS-IF in Japanese adults. This study provides insights into the etiologies and treatment patterns of SBS-IF in Japanese adults. Overall, the characteristics of these Japanese adult patients with SBS-IF receiving PN were similar to those reported for patients with SBS-IF in other parts of the world. Successful weaning from PN was achieved for only a small proportion of patients. Moreover, because of the complexity of SBS-IF, we recommend that intestinal rehabilitation programs be overseen by a multidisciplinary team in a specialized center to improve the treatment outcomes of patients with this distressing condition.

## Supplementary Information

Below is the link to the electronic supplementary material.

Supplementary file1 (PDF 231 KB)

## Data Availability

The data that support the findings of this study are available with permission from MDV under the same conditions as used in this study. Restrictions apply to the availability of these data, which were used under a contract between MDV and Takeda Pharmaceutical Company Limited.
