# Supplementary material for: Etiologies and treatments of chronic intestinal failure-short bowel syndrome (SBS) in Japanese adults: a real-world observational study
Source: Surg Today. 2022 Feb 23;52(9):1350–7. doi: 10.1007/s00595-022-02469-9 (PMC9393151; doi:10.1007/s00595-022-02469-9)
Supplement: Supplementary file 1 — Supplementary file1 (PDF 231 KB) [file 595_2022_2469_MOESM1_ESM.pdf]

Supplementary Appendix

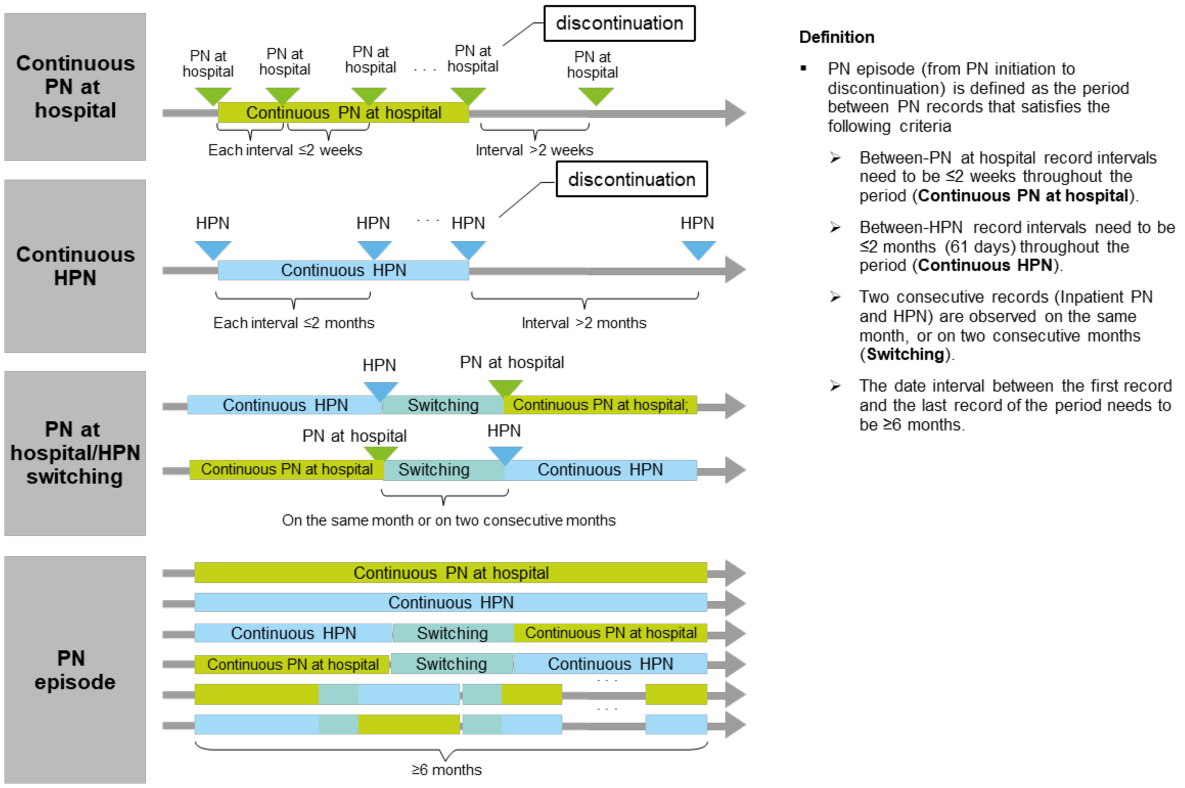

Supplementary Fig. 1 Schematic diagram of the definitions of the various types of parenteral nutrition episode.

*The case of alternately repeating PN at hospital and HPN with switching is also treated as PN episode

HPN home parenteral nutrition, *PN* parenteral nutrition

a

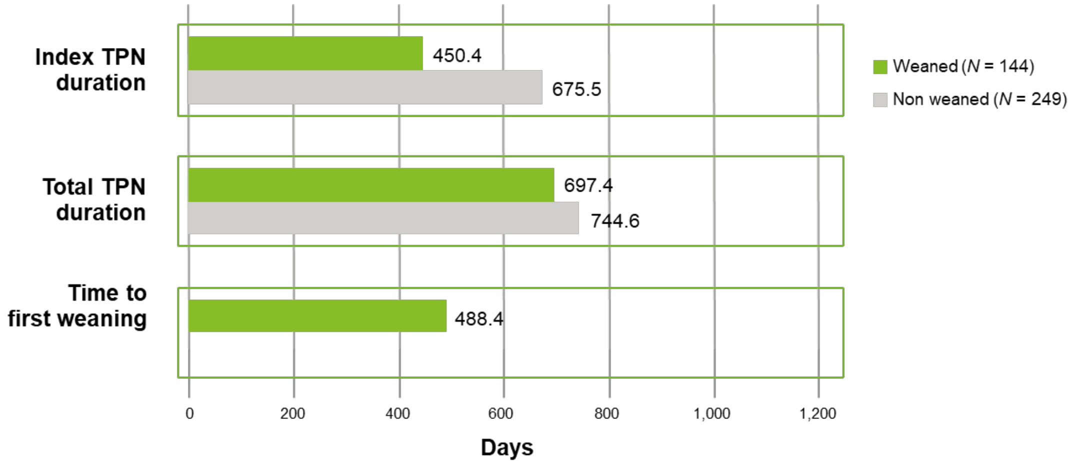

b

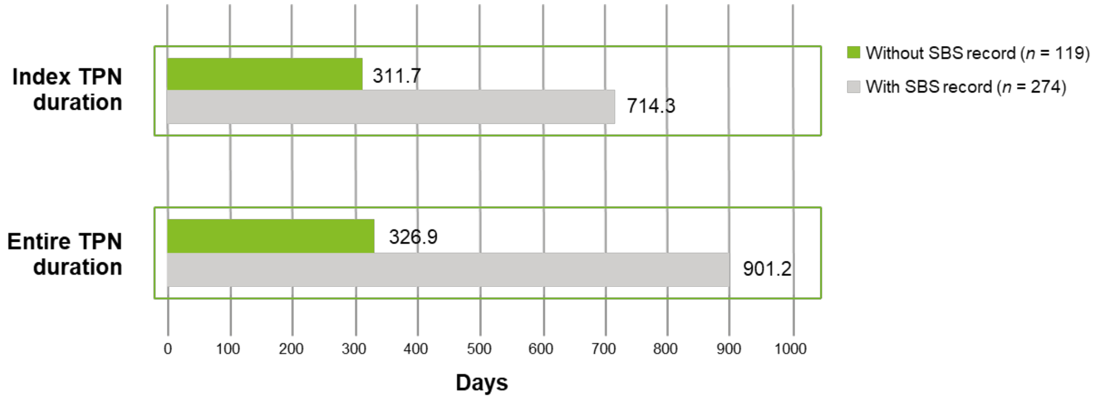

Supplementary Fig. 2 PN duration in weaned and unweaned patients (**a**) and patients with and without an SBS record (**b**)

PN parenteral nutrition, *SBS* short bowel syndrome

Supplementary Table 1 Causative diseases of short bowel syndrome[a]

ICD-10 code	Disease name	*n*	%
K567	Ileus	110	28.0
K509	Crohn's disease	65	16.5
K650	Acute pan-peritonitis	41	10.4
K565	Adhesive ileus	31	7.9
K650	Perforative peritonitis	28	7.1
K562	Strangulation ileus	26	6.6
K631	Small intestinal perforation	26	6.6
K508	Crohn's ileocolitis	24	6.1
K913	Postoperative adhesive ileus	23	5.9
K913	Postoperative ileus	20	5.1
K550	Intestinal necrosis	19	4.8
K566	Small intestinal ileus	18	4.6
K550	Small intestinal necrosis	17	4.3
K632	Small intestinal fistula	17	4.3
K560	Paralytic ileus	16	4.1
T814	Postoperative intra-abdominal abscess	16	4.1
K550	Acute superior mesenteric artery occlusion	14	3.6
K659	Peritonitis	11	2.8
K500	Crohn's enteritis	10	2.5
K550	Thrombosis of mesenteric artery	9	2.3
K566	Obstructive ileus	9	2.3
K567	Subileus	9	2.3
K550	Nonobstructive mesenteric ischemia	8	2.0
K566	Stenosis of small intestine	8	2.0
K650	Localized peritonitis	8	2.0
D126	Familial polyposis coli	7	1.8
K520	Radiation enteritis	6	1.5

K660	Intestinal adhesions	6	1.5
K650	Acute peritonitis	5	1.3
Q431	Hirschsprung disease	5	1.3
A052	Necrotic enteritis	4	1.0
C179	Small intestine carcinoma	4	1.0
K559	Ischemic enteritis	4	1.0
K567	Pseudoileus	4	1.0
K650	Acute circumscribed peritonitis	4	1.0
N735	Pelvis peritonitis	4	1.0
R190	Intraperitoneal mass	4	1.0
C859	Small intestinal malignant lymphoma	3	0.8
D126	Adenomatosis coli	3	0.8
D372	Small intestinal tumor	3	0.8
I728	Superior mesenteric artery dissociation	3	0.8
K501	Crohn’s colitis	3	0.8
K633	Small intestinal ulcer	3	0.8
K650	Intestinal perforation peritonitis	3	0.8
R590	Mesenteric lymph node enlargement	3	0.8
C172	Ileal cancer	2	0.5
D126	Colorectal adenoma	2	0.5
K315	Superior mesenteric artery syndrome	2	0.5
K500	Crohn’s ileitis	2	0.5
K508	Stomach Crohn’s disease	2	0.5
K562	Small bowel volvulus	2	0.5
K566	Mechanical ileus	2	0.5
K650	Perforative intra-abdominal abscess	2	0.5
K658	Bacterial peritonitis	2	0.5
K928	Digestive tract stenosis	2	0.5
A099	Inflammatory bowel disease	1	0.3
C241	Periampullary cancer	1	0.3

C481	Malignant mesenteric tumor	1	0.3
D014	Small intestinal carcinoma in situ	1	0.3
D126	Gardner syndrome	1	0.3
D484	Mesenteric tumor	1	0.3
I728	Superior mesenteric artery aneurysm	1	0.3
K353	Appendiceal peritonitis	1	0.3
K469	Internal hernia	1	0.3
K500	Duodenal Crohn’s disease	1	0.3
K501	Rectal Crohn’s disease	1	0.3
K509	Steroid-dependent Crohn’s disease	1	0.3
K550	Mesenteric vein thrombosis	1	0.3
K550	Acute mesenteric artery occlusion	1	0.3
K550	Acute small intestinal ischemia	1	0.3
K550	Ileal necrosis	1	0.3
K551	Chronic ischemic enteritis	1	0.3
K562	Complex obstructive ileus	1	0.3
K562	Sigmoid volvulus	1	0.3
K571	Small intestine diverticulosis	1	0.3
K650	Duodenal perforative peritonitis	1	0.3
K650	MRSA peritonitis	1	0.3
K650	Celiac pelvis abscess	1	0.3
K658	Biliary peritonitis	1	0.3
K659	Postoperative peritonitis	1	0.3
Q432	Pseudo-Hirschsprung’s disease	1	0.3
Q438	Chronic idiopathic intestinal pseudo-obstruction	1	0.3
Q793	Gastroschisis	1	0.3
R190	Mesenteric mass	1	0.3

[a]84 diseases are listed. Cancers are highlighted in blue.

Supplementary Table 2 Rates of biologic treatment use for Crohn's disease patients (*n* = 79)

Biologic	Lookback period, *n* (%)	Index PN period, *n* (%)	Lookback – follow-up period, *n* (%)
Adalimumab	13 (16.5)	20 (25.3)	28 (35.4)
Infliximab	7 (8.9)	19 (24.1)	24 (30.4)
Ustekinumab	0	5 (6.3)	9 (11.4)
Vedolizumab	0	0	1 (1.3)

PN parenteral nutrition
